# Contrasting physiological responses to excess heat and irradiance in two tropical savanna sedges

**DOI:** 10.1093/aobpla/plt051

**Published:** 2013-11-07

**Authors:** C. John-Bejai, A. D. Farrell, F. M. Cooper, M. P. Oatham

**Affiliations:** Department of Life Sciences, The University of the West Indies, St. Augustine, Trinidad and Tobago

**Keywords:** Canopy temperature depression, cell membrane thermostability, environmental gradient, heat stress, leaf functional traits, leaf reflectance, light stress, tropical savanna.

## Abstract

Tropical hyperseasonal savannas provide a rare example of a tropical climax community dominated by graminoid species. Species living in such savannas are frequently exposed to excess heat and light, in addition to drought and waterlogging, and must possess traits to avoid or tolerate these stress factors. Here we examine the contrasting heat and light stress adaptations of two dominant savanna sedges: *Lagenocarpus guianensis*, which is restricted to the sheltered forest edge, and *Lagenocarpus rigidus*, which extends from the forest edge to the open savanna. An ecotone extending from the forest edge to the open savanna was used to assess differences in a range of physiological traits (efficiency of photosystem II, cell membrane thermostability, stomatal conductance, leaf surface reflectance and canopy temperature depression) and a range of leaf functional traits (length : width ratio, specific leaf area and degree of folding). *Lagenocarpus guianensis* showed significantly less canopy temperature depression than *L. rigidus*, which may explain why this species was restricted to the forest edge. The range of leaf temperatures measured was within the thermal tolerance of *L. guianensis* and allowed photosystem II to function normally, at least within the cool forest edge. The ability of *L. rigidus* to extend into the open savanna was associated with an ability to decouple leaf temperature from ambient temperature combined with enhanced cell membrane thermostability. The high degree of canopy temperature depression seen in *L. rigidus* was not explained by enhanced stomatal conductance or leaf reflectance, but was consistent with a capacity to increase specific leaf area and reduce leaf length: width ratio in the open savanna. Plasticity in leaf functional traits and in cell membrane thermostability are key factors in the ability of this savanna sedge to survive abiotic stress.

## Abstract (precis)

Trinidad's Aripo Savanna is a rare example of an intact tropical grassland. It is a living laboratory in which to explore the mechanisms used by plants to survive the stress of life in the full glare of the equatorial sun. We found that the dominant species, *Lagenocarpus rigidus,* avoids overheating not through higher transpiration or more reflective leaf surfaces (as expected), but by altering the size and shape of its leaves to suit each location. This plasticity in leaf morphology is combined with plasticity in cell membrane properties, which allows the leaves to tolerate periods of extreme heat. In the absence of these traits a closely related species *Lagenocarpus guianensis*, finds its range restricted to the shaded savanna edges where heat and light are less overbearing

## Introduction

As the effects of global climate change become more apparent, there is increasing interest in understanding how plants will respond to a warmer environment (Reynolds *et al.* 2001; Larkindale *et al.* 2005; Allakhverdiev *et al.* 2008; Mittler *et al.* 2012). By understanding the responses of species that have evolved in environments frequently exposed to heat stress, we will be better placed to manage the transition to a warmer environment in both wild and cultivated systems.

Vegetation communities within the Aripo Savannas Environmentally Sensitive Area, in the northeast of Trinidad, are segregated into two distinct habitats: open savanna and seasonally flooded marsh forest (Beard 1953; Richardson 1963). The edaphic savanna provides a rare opportunity to study species traits in a tropical climax community dominated by graminoid and herbaceous species. In particular, it offers an opportunity to better understand the ecophysiology of sedges, an economically important but understudied group (Barrett 2013), which is dominant in this savanna. The abiotic character of the open savanna poses a challenge to plant species, as illustrated by the markedly lower floral diversity of these areas as compared with adjacent marsh forest (Richardson 1963). Resistance to environmental extremes is a dominant selective force in adverse habitats such as this and species capable of persisting will be expected to display adaptive responses to their environment. Resident species must cope with the challenges imposed by edaphic factors, namely poor soil drainage, low fertility and an acidic pH (Richardson 1963). The influence of soil drainage on species composition is observed to be greatest in the open savannas as the shallow clay pan depths that characterize these areas result in more pronounced periods of inundation and subsequent drought (Richardson 1963).

Coupled with the challenges imposed by soil drainage, open savanna species will inevitably be exposed to periods of high irradiance and high temperature. The influence of these two factors on the traits of the open savanna species has yet to be determined. In light of predicted climate change, the adaptive responses of species to high temperature and high irradiance have garnered considerable attention in recent years (Reynolds *et al.* 2001; Larkindale *et al.* 2005; Allakhverdiev *et al.* 2008; Mittler *et al.* 2012). Understanding the response of graminoid species is of particular interest given their importance as agricultural crops (Reynolds *et al.* 2001). Within natural systems, the adaptive response of species to spatial heterogeneity in temperature can be used as a proxy for temporal change in temperature (Rice and Emery 2003; Davis *et al.* 2005; Guerin *et al.* 2012). The Aripo Savannas provide an opportunity to study such responses in a relatively intact climax ecosystem. Furthermore, successful management and restoration of such environments during climate change necessitates that adaptive responses of resident species to microclimate be understood (Rice and Emery 2003; Harris *et al.* 2006; Guerin *et al.* 2012).

A botanical survey of the savanna (Cooper and Oatham 2012) found differences in distribution between two widespread and related sedges: *Lagenocarpus guianensis* and *Lagenocarpus rigidus*, the former limited to the margins of marsh forest and the latter occurring in open savanna as well as at the forest margin. The capacity of *L. rigidus* to inhabit areas of open savanna was taken as indicative of the presence of stress adaptations that are not shared with *L. guianensis*. The measurements conducted in this study aim to compare these two species with respect to the presence of traits related to high irradiance and high temperature in order to better understand how graminoid species respond to prolonged exposure to such factors. First, it was hypothesized that compared with *L. guianensis*, *L. rigidus* within areas of open savanna will exhibit physiological traits expected for plants resistant to high irradiance and high temperature, namely reflective leaf surfaces (Ehleringer and Mooney 1978; Long and Humphries 1994), resistance to membrane disruption at high temperatures (Bajji *et al.* 2002; Allakhverdiev *et al.* 2008), employment of transpirational cooling (Upchurch and Mahan 1988; Burke and Upchurch 1989; Hatfield and Burke 1991; Radin *et al.* 1994; Reynolds-Henne *et al.* 2010), successful canopy temperature depression (Balota *et al.* 1993; Amani *et al.* 1996; Lu *et al.* 1998; Reynolds *et al.* 2001) and a high capacity to protect and repair photosystem II (Liu and Huang 2000; Baker 2008). Second, it was hypothesized that compared with *L. guianensis*, *L. rigidus* within areas of open savanna will exhibit leaf functional traits expected for plants resistant to high irradiance and high temperature, namely low leaf dimensions, low specific leaf area (SLA) (typical of high-light or high-stress environments) and a high degree of leaf folding (Nicotra *et al.* 2011; Guerin *et al.* 2012).

## Methods

### Study site

The study was carried out within Savanna II of the Aripo Savannas Environmentally Sensitive Area over a 2-month period: June 2012 to July 2012. The Aripo Savannas are hyperseasonal savannas (Sarmiento 1983) with a drought season followed by a wet season with associated flooding due to poor soil drainage. Hyperseasonal savannas are distributed in Central America, on the Guiana Shield and in the Cerrado of southern Brazil. Species compositions differ between hyperseasonal savannas in terms of importance of different species but generally the species occurring on these savannas are widespread. The focal species of this study, *L. rigidus* and *L. guianensis*, are found on the Guiana Shield, in the Cerrado of Brazil and Bolivia and also in Central America in the case of *L. guianensis*.

A sampling zone was established, extending from an area of open savanna to an area of sheltered savanna on the margin of adjacent marsh forest. Five sampling zones were demarcated. Each zone extended c. 3 m perpendicular to the forest edge × 10 m parallel to the forest edge. The centre of each zone was 14 m apart from the last. The zones extended from the sheltered marsh forest (0 m) via the transitional savanna zones (14, 28 and 42 m) to the open savanna proper (56 m).

### Characterizing the environmental gradient along the ecotone

Clay pan depth was measured within each sampling zone utilizing an aluminium soil probe. The probe was inserted perpendicular to the soil surface and the depth at which resistance was met was taken to be the depth of the clay pan. Soil moisture was measured as volumetric water content (VWC) using a time-domain reflectometer (TDR 100, Fieldscout, Spectrum Technologies, USA). Six replicate measurements were taken per zone—to account for the microtopography of the savanna, measurements were taken from the elevated humps where the sedges were growing, avoiding the surrounding water-filled hollows. To estimate the degree of shading from the forest canopy within each sampling zone, a plant canopy analyser (LICOR, Lincoln, NE, USA) was employed to obtain leaf area index values on an overcast day, with the sun located directly overhead. A 75 % shield was attached to the device's sensor in order to minimize shading contributed by the researcher. The sensor was oriented to point towards the adjacent swamp forest so as to capture the shading influence of the forest canopy and held at a height of 1.5 m (above the height of the grass canopy). Five dataloggers were placed along the ecotone (Hobo Pendent; Onset Corp., Bourne, MA, USA), one in the centre of each zone, on a single measurement day (during which diurnal measurements of the physiological parameters were taken). The dataloggers were placed at soil level on exposed hump areas.

### Physiological measurements

Collection of leaf material and infield measurements were carried out within each sampling zone, using 10 replicates of each species, unless otherwise stated. All measurements were made using healthy, non-senescent, fully expanded leaves.

Diurnal time courses of stomatal conductance and leaf surface temperature were measured together. Within each sampling zone, stomatal conductance and leaf surface temperature were measured over a diurnal time course. Stomatal conductance of each replicate was taken from the abaxial surface of a single leaf at 0800, 1000, 1200, 1400 and 1700 h with a leaf porometer, avoiding the central vein and degraded areas (SC-1; Decagon Devices, Pullman, WA, USA). Leaf surface temperatures were measured at 0600, 1200 and 1700 h from a single leaf of each replicate using a thermal camera (Toughcam; Infrared Cameras Inc., Beaumont, TX, USA). Distance was set to 0.45 m and emissivity to 0.98. The camera was held at a fixed distance and angle from the leaf for all measurements; during the early morning period, when leaf temperatures were closely coupled to that of the environment, a warm background was placed behind the leaf to increase the contrast of captured images.

Within each sampling zone, a single leaf was collected from each replicate plant and reflectance properties were assessed using a portable leaf spectrometer (CI-710; CID, Camas, WA, USA), calibrated according to the manufacturer's specifications. Measurements were taken from the adaxial leaf surface against a black background. From the data obtained, average percentage reflectance over the 400–700 nm range was determined (Ehleringer and Mooney 1978).

The quantum efficiency of photosystem II (*F*_v_/*F*_m_) was assessed using a portable chlorophyll fluorometer (MINI-PAM; Walz, Effeltrich, Germany). Measurements were made at midday (1100–1300) from leaves that were dark adapted for at least 30 min using leaf clips. Five replicate plants were measured in each zone using a single dark-adapted leaf for each replicate, utilizing the manufacturer's protocol.

Cell membrane thermostability (CMT) was measured by the ion leakage method, with leaf tissue exposed to a 30-min heat shock *in vitro* (Reynolds *et al.* 2001; Rahman *et al.* 2004; Farrell *et al.* 2012). The optimum heat-shock treatment of 50 °C was established after preliminary experiments with all of the samples (30, 35, 45, 50 and 55 °C; data not shown). A single leaf was collected from each replicate plant, placed in an insulated cooler and transported to the laboratory. In the laboratory each leaf was rinsed with deionized water. The central portion of each leaf was cut into 5-mm segments, which were placed in two labelled vials of deionized water (15 mL), with each vial receiving 25 segments. All vials were placed in a shaker for 10 min, subsequent to which the conductivity of each vial was determined using a bench top conductivity probe (PC 700; Oakton, Vernon Hills, IL, USA). Conductivity values obtained after 10 min were averaged for each species; this value was taken as the background conductivity for the species and was subtracted from all values obtained below. The heat-shock vials were placed in a preheated waterbath for 30 min at 50 °C. Subsequent to this the vials were incubated in a shaker for 24 h; the temperature within the shaker was maintained below 30 °C. Following incubation in the shaker, the conductivity of each sampling vial was measured; this value was taken as pre-lysis conductivity. Vials were then autoclaved for 30 min to achieve lysis of leaf material and conductivity was then measured; this value was taken as post-lysis conductivity. Percentage ion leakage for each vial was calculated as: (pre-lysis conductivity/post-lysis conductivity) × 100.

### 

### Plant and leaf functional traits

Plant height was measured in the field as the shortest distance between the uppermost leaves and the soil surface; measurements were made from 25 replicates in each zone (Cornelissen *et al.* 2003).

Leaf material was sampled in accordance with Cornelissen *et al.* (2003) with 10 replicate leaves collected from each zone. Leaf material was collected from each replicate at midday from within each of the sampling zones. Leaves were sealed in polythene bags with moist tissue paper and transported to the laboratory in an insulated box (∼1 h). Leaf length was measured by a ruler as the distance from the ligule to the leaf tip. Leaf width (furled and unfurled) was measured at the widest point using a caliper (Spi 2000; Vernier, FL, USA). Leaf lamina length : width ratio was calculated as: leaf length (ligule to tip)/unfurled width (measured at the widest point). Leaf folding ratio was calculated as: (unfurled width − folded width)/(unfurled width), where 0.0 represents no folding and 0.5 represents a leaf folded completely on one axis.

The SLA was measured from 10 × 1 cm segments taken from the central part of each leaf. Segment area was estimated by multiplying the unfurled leaf width × 1 cm (the length of the segments). The segments were oven dried for 24 h at 70 °C, placed in a room-temperature desiccator for 10–15 min and then weighed (segment dry weight) (XX70 Precision Balance, Denver Instruments, New York, NY, USA). The SLA was calculated for each leaf as (mm^2^ mg^−1^): segment area/segment dry weight.

### Statistical analysis

Differences between species in the sheltered forest zone were tested by one-way ANOVA (*P* < 0.05; Genstat v.15 (VSNI Ltd, Hemel Hempstead, UK)). For *L. rigidus*, differences between zones were tested by one-way ANOVA with multiple comparisons (Bonferroni; *P* < 0.05), or by two-way ANOVA with multiple comparisons (Bonferroni; *P* < 0.05) for variables measured during the diurnal time course.

## Results

### Environmental changes along the ecotone

**Figure 1.**
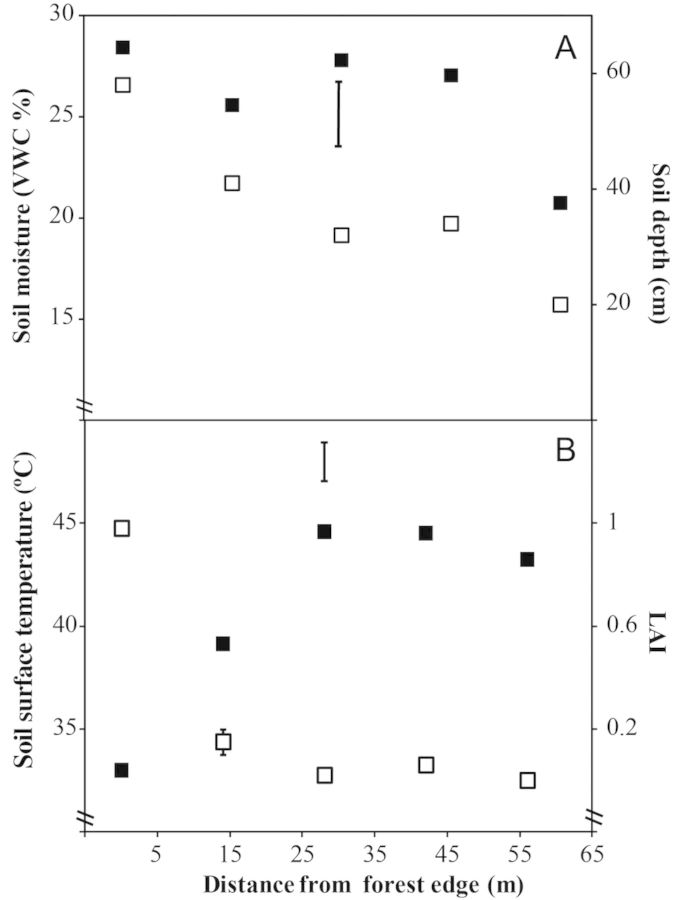
Abiotic factors measured along a savanna ecotone at five distances from the forest edge. (A) Soil moisture content (closed symbols; VWC %) and soil depth (open symbols) measured from a single central point within each sampling zone. VWC data are means of six measurements; the vertical bar indicates l.s.d_0.05_ for VWC. (B) Midday soil surface temperature (closed symbols) and canopy shading (open symbols; LAI). Five LAI measurements were taken at each distance, with the sensor being positioned towards the forest edge and held at a height of 1.5 m (above the height of the grass canopy). Error bars indicate SE for LAI (when larger than the symbol). Soil surface temperature was measured between 1200 and 1230 h in 5-min intervals; data are means of seven time intervals; the vertical bar indicates l.s.d_0.05_ for soil surface temperature.

Environmental gradients were detected for each of the environmental variables investigated along the ecotone. Soil depth was highest at the forest edge (58 cm) and decreased towards the open where it was just 20 cm (Fig. 1A). Soil moisture was highest at the forest edge (VWC of 28 %) and remained high in the transitional zones before dropping markedly in the open savanna proper (56 m from the forest edge, VWC of 20 %) (Fig. 1A). The high soil moisture at the forest edge and in the transitional zones reflects the fact that these areas were inundated with water throughout the study. These areas were made up of saturated humps and water-filled hollows, with the large sedges used in this study restricted to the humps. The shallower soil in the open savanna loses water more quickly as reflected in the lower soil moisture. The forest edge was characterized by the highest canopy shading (highest leaf area index; LAI); there was a marked drop in shading at 14 m from the edge, a smaller drop at 28 m, after which shading remained low throughout the rest of the ecotone (Fig. 1B). Midday soil surface temperature varied significantly along the ecotone (*P* < 0.001). Post hoc testing revealed that a significant increase in temperature accompanied the transition from the forest edge to 14 m and from 14 m to the remaining zones, all of which had similar soil surface temperatures (Fig. 1B).

### Physiological measurements

**Figure 2.**
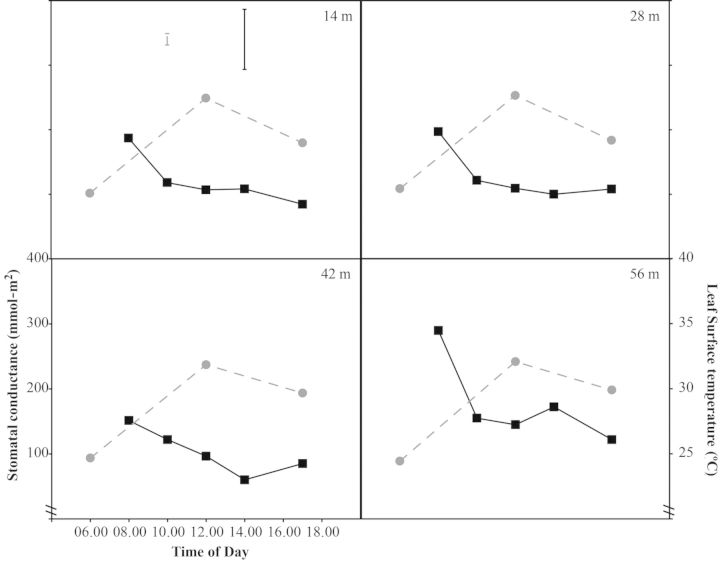
Diurnal time courses for stomatal conductance (squares) and leaf surface temperature (circles) of *L. rigidus* at four distances from the forest edge: 14, 28, 42 and 56 m. Time courses for forest edge plants are shown in Fig. 3. Stomatal conductance and leaf surface temperature were measured at predetermined times over the course of 1 day from a single leaf per replicate plant. Data are means of five replicate plants. Vertical bars indicate l.s.d_0.05_ for distance × time of day for stomatal conductance (solid bar) and leaf surface temperature (shaded bar).

**Figure 3.**
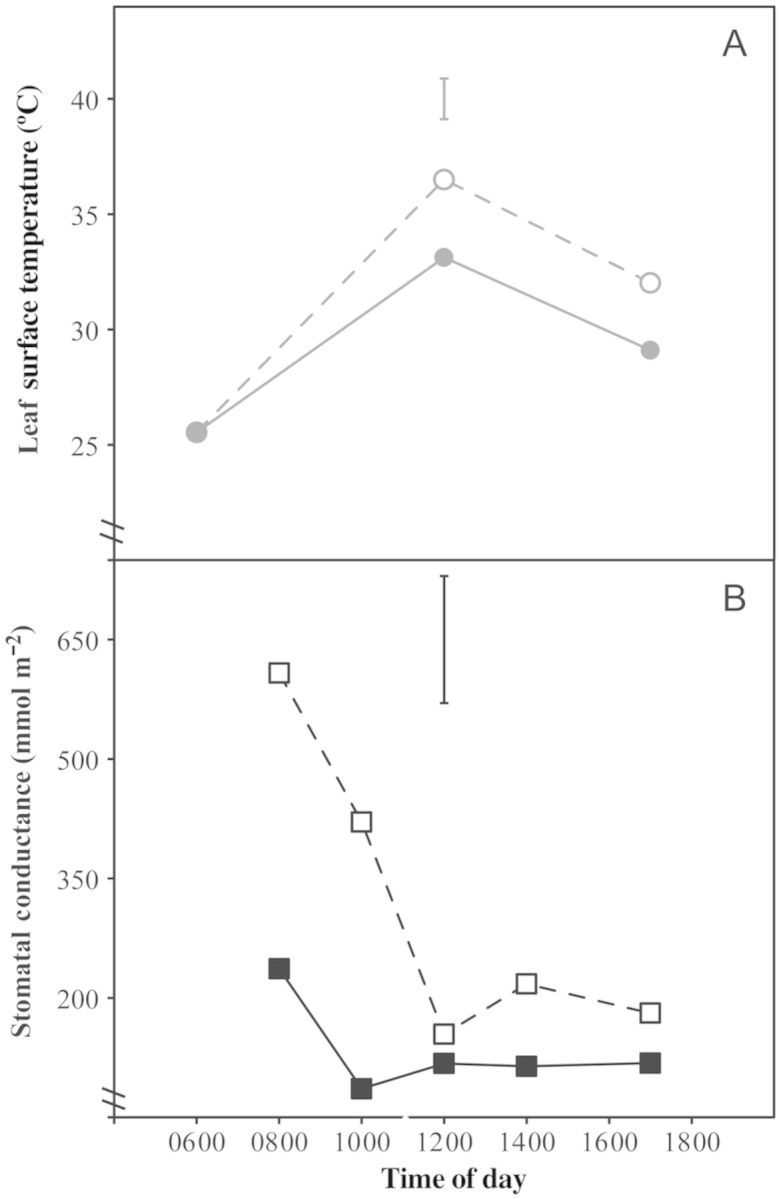
Diurnal time course for (A) leaf surface temperature and (B) stomatal conductance of *L. rigidus* (closed symbols) and *L. guianensis* (open symbols) at the forest edge (0 m). Stomatal conductance and leaf surface temperature were measured at predetermined times over the course of 1 day from a single leaf per replicate plant. Data are means of five replicate plants. Vertical bars indicate l.s.d_0.05_ for time of day × species for stomatal conductance (solid bar) and leaf surface temperature (shaded bar).

**Figure 4.**
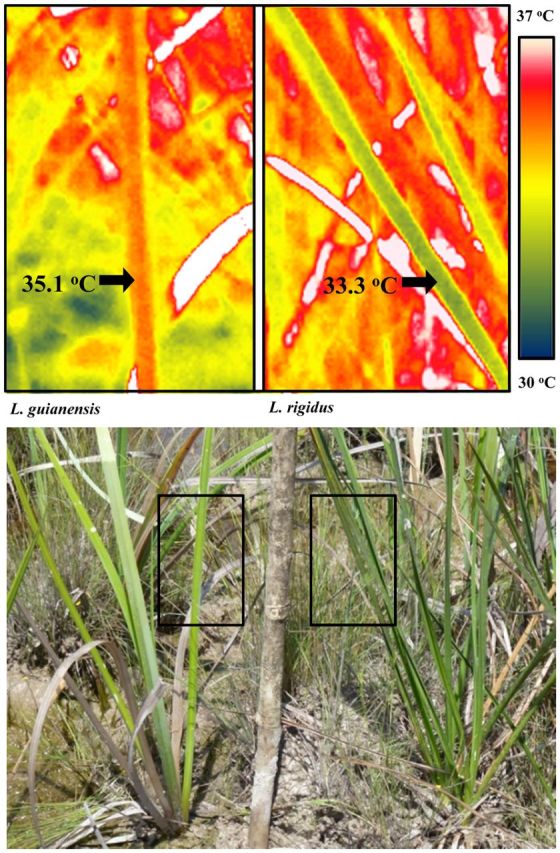
Thermal images showing midday leaf surface temperature of *L. guianensis* (left) and *L. rigidus* (right) growing in close proximity at the forest edge. The accompanying photograph indicates the areas used for the thermal images. The white areas in the thermal image represent dead leaves that are above the maximum temperature range. Similar images were used to measure the leaf surface temperatures shown in Fig. 3.

Changes in stomatal conductance and leaf surface temperature followed a similar diurnal pattern for both species. In all cases, leaf surface temperatures were significantly different at each time point (*P* < 0.001): temperatures were lowest during the morning period, higher at midday and transitional in the afternoon (Figs 2 and 3). For *L. rigidus*, there was no overall association between leaf surface temperature and distance from the forest edge (*P* > 0.05). There was a significant interaction between time point × distance (*P* < 0.05), with the final two zones (44 and 56 m) showing less cooling in the afternoon. In all cases, stomatal conductance was highest at 0800 and progressively decreased over the course of the day (Figs 2 and 3). For *L. rigidus*, distance from the forest edge had a significant impact on stomatal conductance (*P* < 0.05), with the highest rates seen in the final zone (56 m) throughout the day (Fig. 2). At the forest edge it was evident that the two species differed significantly with respect to leaf temperature, with *L. rigidus* having significantly cooler leaves at midday and in the afternoon (*P*< 0.001; Figs 3 and 4). Transpiration also differed between species, with *L. guianensis* having higher stomatal conductance throughout, significantly so at 0800 and 1000 (*P* < 0.001).

**Figure 5.**
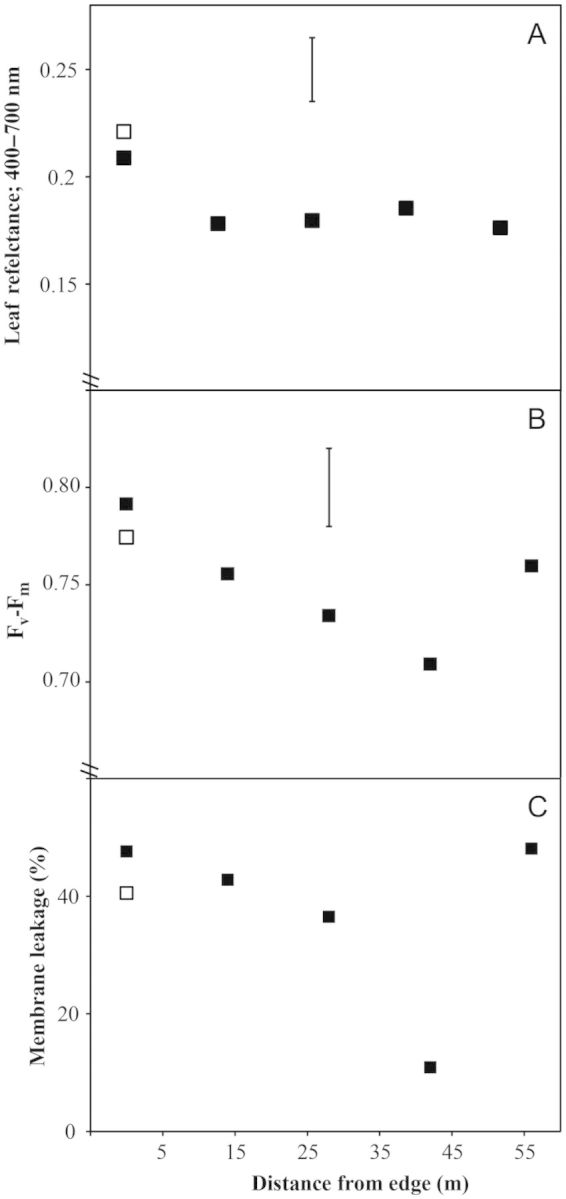
Physiological parameters measured from *L. rigidus* (closed symbols) and *L. guianensis* (open symbol) growing along a savanna ecotone at five distances from the forest edge. (A) Average leaf reflectance (400–700 nm); (B) quantum efficiency of photosystem II (*F*_v_*/F*_m_) measured at midday from leaves that were dark adapted for 30 min; and (C) membrane leakage of leaf segments exposed to 50 °C for 1 h (data are percentages of membrane leakage following full lysis). Data are means of 10 replicate plants. No significant differences were found between *L. guianensis* and *L. rigidus* at the forest edge for any parameter (*P* > 0.05). The vertical bar indicates l.s.d_0.05_ for *L. rigidus* × distance.

No significant difference in reflective capacity was detected between the two species at the forest edge (*P* > 0.05) (Fig. 5A). Similarly no significant variation in reflective capacity was detected upon comparing *L. rigidus* individuals in relation to distance from the forest edge (*P* > 0.05) although the reflectance was higher in the sheltered forest zone (Fig. 5A).

The quantum efficiency of photosystem II for *L. guianensis* and *L. rigidus* was similar at the forest edge (*P*> 0.05), with both species exhibiting *F*_v_/*F*_m_ values within the range expected for healthy plants (Liu and Huang 2000). The *F*_v_*/F*_m_ of *L. rigidus* varied significantly in relation to distance from the forest edge (*P* < 0.01) (Fig. 5B). *Post hoc* testing revealed that *F*_v_*/F*_m_ was significantly lower for individuals at 42 m as compared with those at the forest edge, with the *F*_v_*/F*_m_ values at 14 and 28 m being transitional. An increase in *F*_v_*/F*_m_ accompanied the transition from 42 to 56 m.

There was no significant difference in the CMT of *L. guianensis* and *L. rigidus* in the sheltered forest zone (*P* > 0.05; Fig. 5C). For *L. rigidus*, exposure to 50 °C resulted in high ion leakage in the sheltered forest zone, becoming gradually lower across the transition zone, significantly so at 42 m and then rising again in the open savanna proper (*P* < 0.01; Fig. 5C).

### Plant and leaf functional traits

**Figure 6.**
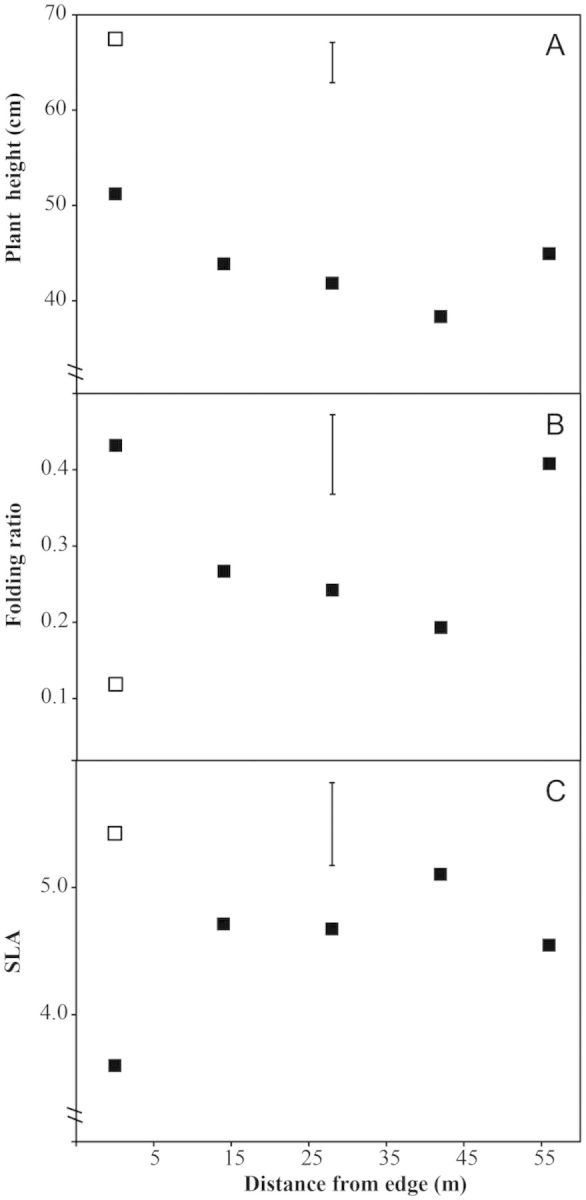
Plant functional traits measured from *L. rigidus* (closed symbols) and *L. guianensis* (open symbol) growing along a savanna ecotone at five distances from the forest edge. (A) Plant height; (B) leaf folding ratio calculated as (unfurled width − folded width)/unfurled width, where higher numbers indicate greater folding; and (C) SLA (mm^2^ mg^−1^). Data are means of 10 replicate plants, except for plant height with 25 replicates. Significant differences were found between *L. guianensis* and *L. rigidus* individuals at the forest edge for all parameters (*P* < 0.001; *P* < 0.001; *P* < 0.01, respectively). The vertical bar indicates l.s.d_0.05_ for *L. rigidus* × distance.

*Lagenocarpus rigidus* plants were tallest in the sheltered forest zone, becoming gradually shorter across the transition zone and then increasing in the open savanna proper (*P* < 0.001; Fig. 6A). *Lagenocarpus guianensis* was significantly taller than *L. rigidus* in the sheltered savanna (*P* < 0.001; Fig. 6A).

**Figure 7.**
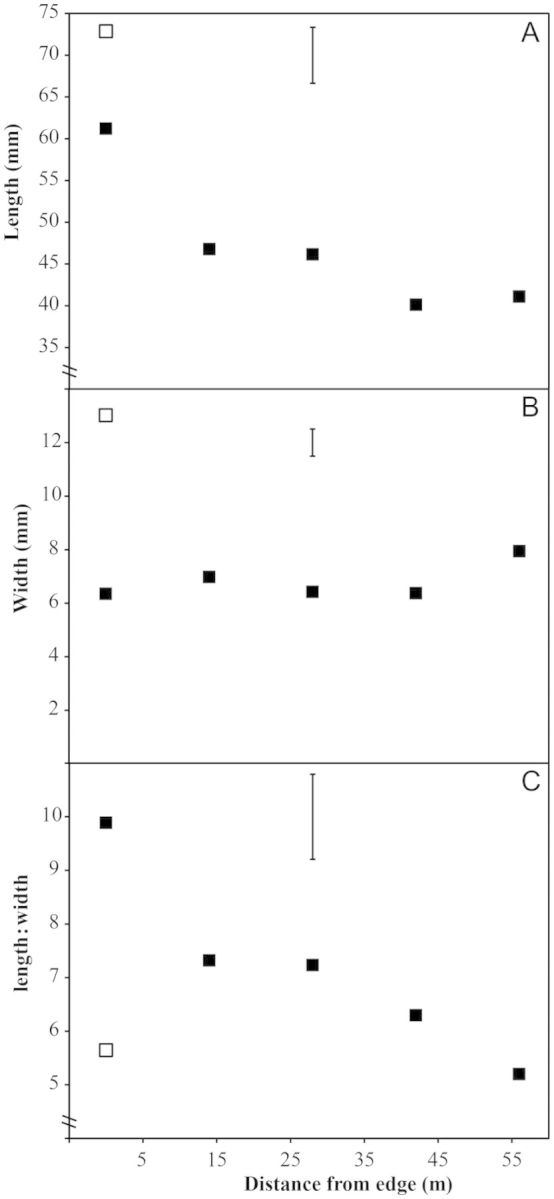
Leaf dimensions measured from *L. rigidus* (closed symbols) and *L. guianensis* (open symbol) growing along a savanna ecotone at five distances from the forest edge. (A) Leaf length, (B) leaf width and (C) leaf length : width r atio. Data are means of 10 replicate plants. Significant differences were found between *L. guianensis* and *L. rigidus* individuals at the forest edge for all parameters (*P* < 0.001). The vertical bar indicates l.s.d_0.05_ for *L. rigidus* × distance.

*Lagenocarpus guianensis* had significantly longer and wider leaves than *L. rigidus* in the sheltered savanna (*P* < 0.001; Fig. 7). *Lagenocarpus rigidus* leaf length was highest in the sheltered forest zone, declining gradually towards the open savanna (*P*< 0.05; Fig. 7A). *Lagenocarpus rigidus* leaf width was constant across the ecotone, apart from a small increase in the open savanna proper. In the sheltered forest zone, the length : width ratio was higher in *L. rigidus* compared with *L. guianensis*, but it fell steadily with distance from the forest edge and was significantly lower in the open savanna (*P*< 0.001; Fig. 7C).

*Lagenocarpus rigidus* leaves generally showed some folding, with the leaf angled at the midrib forming a V-shaped profile. *Lagenocarpus guianensis* leaves had a distinct W-shaped profile with no true folding observed. The degree of leaf folding in *L. rigidus* again followed a ‘U-shaped’ pattern with folding highest in the sheltered forest zone then declining gradually and rising in the open savanna proper (*P* < 0.001; Fig. 6B).

For *L. rigidus*, leaf dry weight followed the U-shaped pattern (data not shown). *Lagenocarpus rigidus* SLA was significantly lower in the sheltered forest zone (3.6) than in the open areas (ranging from 4.5 to 5.1; *P*< 0.001; Fig. 6C). In the open, *L. rigidus* SLA shows no clear pattern. *Lagenocarpus guianensis* SLA (5.4) was significantly higher than that of *L. rigidus* in the sheltered forest zone (*P*< 0.01), but similar to the *L. rigidus* SLA in the open areas.

## Discussion

The open savanna emerged from the shade at ∼28 m from the forest edge. This stressful habitat was characterized by high temperature and high irradiance. *Lagenocarpus rigidus* midday leaf temperatures were maintained within a relatively narrow range irrespective of proximity to the forest edge (Figs 2 and 3). Given the distinct thermal character of the transitional zones and open savanna proper, as compared with the forest edge, this observation provides evidence that *L. rigidus* possesses a high capacity for canopy temperature depression. This high capacity for canopy temperature depression is also evident where the two species grow in close proximity, with *L. rigidus* maintaining a lower leaf temperature (Fig. 4). Canopy temperature depression has been shown to be effective in preventing the onset of thermal stress in graminaceous crops (Reynolds *et al.* 2001), as well as other crop species (Upchurch and Mahan 1988; Hatfield and Burke 1991). The leaf temperature profiles of *L. rigidus* suggest that the species' capacity to inhabit areas of open savanna is partially mediated by avoidance of thermal stress. Leaf temperatures of *L. guianensis* were observed to be more closely coupled to that of the environment, with leaves heating up substantially by midday (Fig. 3A). In both cultivated and non-cultivated species, canopy temperature depression has been observed to be positively correlated with stomatal conductance (Burke and Upchurch 1989; Radin *et al.* 1994; Amani *et al.* 1996; Lu *et al.* 1998; Nagler *et al.* 2003); the lower potential of *L. guianensis* to achieve canopy temperature depression may in part be attributable to the rapid reduction in stomatal conductance observed during the morning period, 0800–1200, although the absolute rate was still similar to that of *L. rigidus* (Fig. 3B). Taking into consideration the inundated conditions that characterize the study site, the diurnal patterns in stomatal conductance observed for *L. guianensis* suggest that the species is isohydric, with midday reductions in stomatal conductance observed irrespective of soil water status (Tardieu and Simonneau 1998; Franks *et al.* 2007).

Highly reflective leaf surfaces reduce the impact of excess irradiance and help to keep leaves cool in environments characterized by high irradiance and high temperature (Pearman 1966; Ehleringer and Bjorkman 1978; Ehleringer and Mooney 1978). However, differences in reflective capacity do not explain the differences in canopy temperature depression between *L. rigidus* and *L. guianensis*, nor do they explain the ability of *L. rigidus* to extend into the open savanna. Plasticity in CMT is a key trait in conferring tolerance to excess heat. Restricted membrane fluidity is essential if cells are to retain their basic functions following exposure to very high temperatures (typically >40 °C tissue temperature), just as maintaining high fluidity is advantageous when cells are exposed to low temperature (Larkindale *et al.* 2005; Farrell *et al.* 2006; Penfield 2008). Measuring the leakage of ions from tissue after exposure to high temperature for a fixed time is a well-established method of comparing thermal tolerance (Reynolds *et al.* 2001; Rahman *et al.* 2004). The method has been applied in agronomic grassland systems (Marcum 1998; Liu and Huang 2000), but has not been widely used in natural ecosystems. Here, both species were seen to have some tolerance to high temperature as membrane leakage was still below 50 % after exposure to 50 °C, a relatively high heat-shock temperature. *Lagenocarpus rigidus* showed an ability to alter its CMT, with leaves in the transitional zones retaining control of membrane permeability after exposure to 50 °C. This observation combined with the low *F*_v_*/F*_m_ values measured in the transitional zones suggests that these leaves were subjected to high temperature in the recent past and that the cell membranes have altered their composition to better withstand heat. At 56 m from the forest, the CMT was lost with membrane leakage similar to that in the sheltered forest zone. This loss of CMT is coincident with a recovery in *F*_v_*/F*_m_. Together, these observations suggest that the plants in the open savanna proper are less subject to high-temperature stress than the plants in the transitional zone. It is not clear what factor allows for a reduction in maximum leaf temperature in the open savanna proper, although the combination of greatly reduced leaf dimensions with a high leaf folding ratio could offer an explanation (see below).

In spite of *L. guianensis*' significantly higher leaf temperatures, the *F*_v_*/F*_m_ of the species did not differ significantly from that of *L. rigidus* at the forest edge (Fig. 5B), with both falling within the range expected for healthy plants (Liu and Huang 2000; Baker 2008). The absence of any detectable impairment in the photosynthetic capacity of *L. guianensis* suggests that the maximum leaf temperatures occurring in the sheltered forest zone were within the thermal tolerance of this species. This is supported by the CMT measurements as membrane permeability was still partially functioning following exposure to 50 °C, well above the range of leaf temperatures experienced in the sheltered forest edge.

The *F*_v_*/F*_m_ values for *L. rigidus* individuals outside of the sheltered forest zone can be taken as indicative of the onset of some form of abiotic stress (Liu and Huang 2000; Li *et al.* 2004; Baker 2008). The *F*_v_*/F*_m_ values corresponding to these areas were markedly lower than that reported by Ludlow *et al.* (1988) for unstressed graminoids and similar to those reported by Lu *et al.* (2004) for non-adapted graminoids growing in a desert environment. Given that leaf temperatures were not found to vary significantly in relation to sampling zone, the reduced *F*_v_*/F*_m_ values observed for *L. rigidus* individuals inhabiting areas away from the forest edge may in part be attributable to the onset of light stress. In the transition zone the excess light was combined with waterlogging, which may explain the lower *F*_v_*/F*_m_ values obtained in these zones compared with the open savanna proper. This pattern mirrors that observed for certain morphological characteristics, i.e. plant height and leaf folding ratio, therein suggesting a correlation between these parameters and physiological status.

*Lagenocarpus rigidus* plants at either end of the ecotone, sheltered and open savanna proper, showed the least amount of stress as evident by the optimal *F*_v_*/F*_m_ values. These plants were taller with leaves that were larger, more folded and had a lower SLA. The generally reduced leaf dimensions and higher SLA in the transitional zones may reflect the impact of combined stresses (heat, excess light and waterlogging). All plots showed clear leaf folding as would be expected in a stressful high-irradiance environment (Ryel *et al.* 1993; Pugnaire and Haase 1996). The high degree of folding in the sheltered forest zone is expected given the greater need for mechanical support in these longer leaves (King *et al.* 1996). The increased folding in the open savanna proper may also have a structural role, or it may reflect a period of water stress in the recent past (Pugnaire *et al.* 1996). Structural properties such as leaf folding, pubescence and leaf thickness are observed to influence leaf reflectance properties (Ehleringer *et al.* 1981; Sims and Gamon 2002). The higher degree of leaf folding observed for *L. rigidus* individuals at the two extremes of the ecotone likely gave them a greater capacity to dissipate excess irradiance through canopy reflectance. The contribution of canopy characteristics such as leaf folding is not detectable using leaf-level reflectance, as measured here, but could be discerned through measurements of canopy-level reflectance (Asner *et al.* 1998). The sheltered zone on the fringe of the marsh forest still received high irradiance, with the forest having a relatively low LAI. As such, this zone cannot be characterized as a light-limited environment, but rather as an environment sheltered from the full impact of excess irradiance. In the sheltered forest zone, *L. rigidus* leaves were larger, more folded and had a lower SLA than in the open. A reduced SLA is not a typical response to a low-light environment, but may reflect the need for increased thickness to support the larger leaves found in this zone (overall leaf length was negatively correlated with SLA; data not shown). All of the SLA values are in the lower end of the range seen in graminoids, placing these sedges among the sclerophyllous species, such as *Lepidosperma* or *Stipa*, rather than the fast-growing tender-leaved grasses (Armas and Pugnaire 2005; Poorter *et al.* 2009).

All leaves were elliptic with length : width ratios >3, but were relatively broad for graminoid species (Traiser *et al.* 2005). *Lagenocarpus rigidus* showed a clear reduction in leaf length and in leaf length: width ratio with distance from the forest edge. Previous studies have shown that within-species leaf size is generally reduced in areas with higher temperature in both graminoid and broadleaved species (Byars *et al.* 2007; Guerin *et al.* 2012 and references therein), although more complex patterns are seen between species (Nicotra *et al.* 2011, 2012). There is an increasing awareness of the importance of phenotypic plasticity in plant functional traits for determining the response of plants to rapid climate change (Nicotra *et al.* 2010). The ability of *L. rigidus* leaves to reduce leaf dimensions is likely a key factor in allowing its range to extend into the exposed savanna areas. Although *L. rigidus* exhibits considerable phenotypic plasticity, the *F*_v_*/F*_m_ values suggest that this species is near the edge of its physiological tolerance, and as such this species is especially vulnerable to rapid climate change. The need to maintain a continuous provision of suitable microclimates should be incorporated into future management plans for the savanna (Rice and Emery 2003; Harris *et al.* 2006).

## Conclusions

The morphology and physiology of the two species growing at the forest edge show this to be a less stressful habitat than the unsheltered savanna. *Lagenocarpus guianensis*, which only occurs in the sheltered forest zone, showed no signs of stress and had longer, wider leaves with higher rates of stomatal conductance but a low capacity for canopy temperature depression and low membrane thermostability. The ability of *L. rigidus* to extend to the open savanna was associated with a relatively high capacity for canopy temperature depression and high membrane thermostability. The high degree of canopy temperature depression seen in *L. rigidus* was not explained by enhanced stomatal conductance or leaf reflectance, but was consistent with a capacity to reduce leaf dimensions in the open savanna.

## Sources of Funding

This work was supported by The Government of the Republic of Trinidad and Tobago Green Fund Project Number GF120000401 and by The University of the West Indies Campus Research and Publication Fund.

## Contributions by the Authors

A.D.F. was the principal investigator and supervisor of C.J. A.D.F. and C.J. oversaw the collection of data in the field and laboratory, performed data analysis and drafted the manuscript. F.M.C. and M.P.O. were involved in site selection and experimental design. All authors helped to draft the manuscript and read and approved the final manuscript.

## Conflicts of Interest Statement

None declared.
